# Correlation and efficacy of TACE combined with lenvatinib plus PD‐1 inhibitor in the treatment of hepatocellular carcinoma with portal vein tumor thrombus based on immunological features

**DOI:** 10.1002/cam4.5841

**Published:** 2023-03-23

**Authors:** Xinhua Zou, Qingyu Xu, Ran You, Guowen Yin

**Affiliations:** ^1^ Department of Tumor Interventional Therapy Jiangsu Cancer Hospital & Jiangsu Institute of Cancer Research & The Affiliated Cancer Hospital of Nanjing Medical University Nanjing City China

**Keywords:** hepatocellular carcinoma, inflammatory cytokines, lenvatinib, programmed cell death‐1 inhibitor, transarterial chemoembolization

## Abstract

**Background:**

Although the appearance of portal vein tumor thrombus (PVTT) is significantly associated with unfavorable prognosis, there is insufficient evidence to confirm the efficacy and safety of the triple combination of transarterial chemoembolization (TACE), lenvatinib, and programmed cell death‐1 (PD‐1) inhibitor for patients with hepatocellular carcinoma (HCC) and PVTT. Furthermore, it remains unclear which patient type can obtain the best survival benefit from this combination therapy.

**Methods:**

The data of 160 patients with HCC and PVTT treated with TACE combined with lenvatinib plus PD‐1 inhibitor (TACE+LEN + PD‐1 group) or TACE combined with lenvatinib (TACE+LEN group) were retrospectively collected and analyzed. To estimate the efficacy and safety of combination therapy for patients with advanced HCC, tumor response, progression‐free survival (PFS), overall survival (OS), biochemical indices, and adverse events (AEs) were assessed in this study. More importantly, tumor immune‐related cytokines were used to identify biomarkers predicting the therapeutic response of combination therapy.

**Results:**

TACE+LEN + PD‐1 was superior to TACE+LEN in OS (23.5 vs. 18.3 months, *p* = 0.0002) and PFS (7.5 vs. 4.3 months, *p* < 0.0001). Moreover, TACE+LEN + PD‐1 achieved more preferable benefits with respect to disease control rate (80.00% vs. 56.67%) and objective response rate (38.57% vs. 24.45%) compared with TACE+LEN in patients with HCC and PVTT (*p* = 0.025). Multivariate analysis showed that Child–Pugh grade, PVTT classification, treatment option, and interleukin (IL)‐6, IL‐17, interferon (IFN)‐α, and vascular endothelial growth factor (VEGF) levels were independent factors related to OS, whereas PVTT classification, treatment option, and IL‐6 and IFN‐α levels were independent factors related to PFS. Furthermore, the subgroup analysis illustrated that the inflammatory cytokines VEGF, IL‐6, IL‐17, and IFN‐α might be novel biomarkers for predicting the survival prognosis of patients with advanced HCC and PVTT treated with TACE+LEN + PD‐1. The safety in the combination group was acceptable.

**Conclusions:**

Compared with TACE+LEN, the triple combination treatment of TACE+LEN + PD‐1 has more promising clinical outcomes and acceptable safety in patients with HCC and PVTT. Child–Pugh grade, PVTT classification, and IL‐6, IL‐17, IFN‐α, and VEGF levels are independent prognostic factors for survival time.

## Graphical abstract

We conducted this retrospective cohort study including 160 patients with HCC and PVTT who received TACE+LEN+PD‐1 or TACE+LEN treatment, and compared clinical outcomes between two groups. We found that TACE+LEN+PD‐1 has markedly better outcomes than TACE+LEN. Moreover, inflammatory cytokines, including IL‐6, IL‐17, VEGF, and IFN‐α, might be novel biomarkers for predicting the survival prognosis of patients receiving TACE+LEN+PD‐1 treatment.

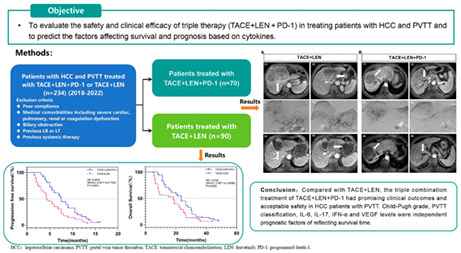

## 1 INTRODUCTION

Hepatocellular carcinoma (HCC) is considered a fatal and common malignant tumor, with a majority of patients with HCC at the intermediate‐advanced stages (Barcelona Clinic Liver Cancer [BCLC] stages B and C), which are unsuitable candidates for ablation, resection, and transplantation.^1^, ^2^ In patients with intermediate‐advanced stage HCCs, although transarterial chemoembolization (TACE) and systemic therapy have been considered effective and standard strategies, these methods lead to poor prognosis and limited median survival time, especially for patients with HCC and portal vein tumor thrombus (PVTT).^3^, ^4^, ^5^, ^6^

PVTT can occur in approximately 35%–55% of patients with HCC, of which 15%–30% involves the main portal vein when diagnosed.^7^ The appearance of PVTT is significantly related to poor prognosis in patients with HCC, which results in portal hypertension, variceal bleeding, and hepatic ischemic damage, causing liver failure and ultimately resulting in 2.7‐month survival period for these patients.^8^ According to the BCLC staging standard, patients with HCC and PVTT are classified as having BCLC stage C. Sorafenib and lenvatinib, which are tyrosine kinase inhibitors (TKIs), were recommended as first‐line treatments for advanced HCC in the SHARP and REFLECT trials, respectively.^9^, ^10^ A previous trial demonstrated that the mean overall survival (OS) periods for advanced HCC in the lenvatinib and sorafenib groups were 13.6 and 12.3 months, respectively. This trial also reported that lenvatinib had similar OS period and acceptable safety as sorafenib.^10^ However, several trials have demonstrated that sorafenib or lenvatinib alone has unsatisfactory efficacy in survival outcomes,^11^, ^12^ whereas a combination of TACE with systemic therapy has shown significantly better outcomes.^13^, ^14^, ^15^, ^16^ In the LAUNCH trial, the combination of lenvatinib and TACE had better median OS (mOS) (17.8 months vs. 11.5 months; hazard ratio [HR], 0.45; 95% confidence interval [CI], 0.33–0.61; *p* < 0.001) and median progression‐free survival (mPFS) (10.6 months vs. 6.4 months; HR, 0.36; 95% CI, 0.27–0.49; *p* < 0.001) compared with lenvatinib monotherapy.^17^

Guidelines recommend that TACE be considered an effective treatment for advanced HCC.^2^, ^5^, ^18^, ^19^, ^20^, ^21^, ^22^ The antitumor mechanism of TACE involves the reduction of tumor size by blocking blood to achieve a therapeutic effect.^23^, ^24^ However, hypoxia induced by TACE treatment can upregulate vascular endothelial growth factor (VEGF) and platelet‐derived growth factor (PDGF), promoting tumor growth and metastasis.^25^, ^26^ Lenvatinib can effectively inhibit VEGF and PDGF receptors to block the formation of tumor neovascularization and control tumor growth. Therefore, the combination of TACE with lenvatinib is significantly effective and safe in treating patients with advanced HCC cases (BCLC C), further improving outcomes in the setting of VP1‐4 PVTT.^27^ Therefore, owing to the poor prognosis and limited survival time in patients with HCC and PVTT, various comprehensive treatments based on TACE may have better survival benefits.

Recently, in the IMbrave150 trial,^28^ the combination of atezolizumab and bevacizumab (Atezo/Bev) achieved significantly better outcomes for the first time compared with sorafenib alone, which has attracted concern about the effectiveness of the combined immunotherapy strategy of programmed cell death‐1 (PD‐1)/programmed death ligand 1 (PD‐L1) and VEGF inhibitors. This combined therapy has antitumor activity, and VEGF inhibitors reprogram the immunosuppressive microenvironment into an immunostimulatory microenvironment. Moreover, the administration of PD‐1/PD‐L1 inhibitors boosts the antitumor activity of T cells.^29^ Although the primary survival end point was not reached only with anti‐PD‐1/PD‐L1 in previous phase III trials,^30^, ^31^ one of the promising outcomes in the combined strategy was the significantly prolonged OS and PFS.^28^, ^32^ In addition to Atezo/Bev, other combinations, especially the combination of pembrolizumab and lenvatinib in a phase Ib trial, have achieved promising clinical outcomes for unresectable HCC.^33^, ^34^, ^35^, ^36^, ^37^ TACE+LEN + PD‐1 compared with TACE alone contributes to a promising therapeutic strategy associated with tumor response and survival time in unresectable HCC.^34^, ^35^, ^36^, ^37^ PVTT is divided into five grades according to the degree of tumor involvement: VP0, VP1, VP2, VP3, and VP4.^38^ Although the Atezo/Bev combination has been considered a first‐line therapy in patients with HCC and PVTT, the efficacy of TACE+LEN + PD‐1 in patients with HCC and PVTT, especially for VP4, has not been confirmed in multicenter studies, and which type of patients can obtain the best survival benefit remains a practical problem that needs to be solved urgently in clinical practice.

The molecular processes underlying hepatocarcinogenesis have been extensively explored in HCC models and patients. The serum levels of interleukin (IL)‐6 and VEGF, which are inflammatory cytokines, are associated with an increased risk of HCC progression,^39^, ^40^, ^41^ suggesting that cytokine signaling pathways are correlated with hepatocellular carcinogenesis. In addition, the high expression of PD‐1 on CD8^+^ T cells^42^ and the high proportion of tumor infiltration of PD‐1^+^ CD8^+^ T cells are involved in tumor progression.^43^ PD‐L1 expression is also an indicator of tumor recurrence in patients with HCC and is associated with tumor invasiveness.^44^, ^45^ Similarly, the interferon (IFN)‐α/γ‐signal transducer and activator of transcription 1‐PD‐L1 axis are essential for regulating the hyporesponsiveness of T cells and the inactivation of liver‐infiltrating T cells in the tumor microenvironment.^46^ In conclusion, it seems to emphasize the significant role of cytokine signaling pathways in the occurrence and development of HCC. Whether the cytokine signaling pathways may be used as markers to determine which type of patients can obtain the best survival benefit from combination therapy remains unknown. This study aimed to evaluate the safety and clinical efficacy of triple therapy (TACE+LEN + PD‐1) in treating patients with HCC and PVTT and to predict the factors affecting survival and prognosis based on cytokines, providing a rational basis for personalized treatment.

## 2 MATERIALS AND METHODS

### 2.1 Study sample

Data from 160 patients with HCC and PVTT admitted to the Department of Interventional Medicine at our institution from February 2018 to May 2022 were retrospectively collected and analyzed. Patients (*n* = 70) who received TACE combined with lenvatinib and PD‐1 inhibitor treatment were classified into the TACE+LEN + PD‐1 group, whereas those who received TACE combined with lenvatinib treatment (*n* = 90) belonged to the TACE+LEN group. HCC was diagnosed based on the following: liver biopsy or serum alpha‐fetoprotein (AFP) ≥400 μg/L (computed tomography [CT] or magnetic resonance imaging [MRI] examination) and AFP <400 μg/L (CT and MRI examination). The inclusion criteria included the following: age ≥ 18 years, more than one measurable lesion according to the modified Response Evaluation Criteria In Solid Tumors (mRECIST) criteria, Child–Pugh A/B, BCLC stage C with PVTT (VP2–4), Eastern Cooperative Oncology Group Performance Status (ECOG‐PS) score <2, and life expectancy ≥8 weeks. The exclusion criteria for this study were as follows: (1) previous liver tumor resection or liver transplantation; (2) poor patient compliance (such as failing to visit the clinic as scheduled, leading to incomplete data); (3) medical comorbidities, including severe cardiac, pulmonary, renal, or coagulation dysfunction; (4) previous treatment with other targeted drugs or PD‐1 immunotherapy; and (5) complications with biliary obstruction.

### 2.2 Treatment procedures

In TACE, femoral artery puncture was performed using the Seldinger method, and the liver tumor‐feeding arteries and the presence or absence of arteriovenous fistula were determined using digital subtraction angiography and then intubated to the tumor‐feeding branches. Patients received either conventional TACE or drug‐eluting bead TACE according to their own preference. Drug‐loaded microspheres or iodized oil containing 40 mg epirubicin was injected to embolize the tumor vessels. TACE was repeated “on demand” upon the demonstration of a viable tumor by follow‐up contrast‐enhanced CT or MRI in patients without deteriorated performance status or organ function. Both groups were orally administered lenvatinib (initial dose, 8 mg/day [<60 kg] or 12 mg/day [≥60 kg]) within 3 days before or after the first TACE treatment, whereas in the TACE+LEN + PD‐1 group, PD‐1 inhibitor (pembrolizumab; Keytruda, Merck & Co., Inc.; initial dose, 200 mg, every 3 weeks), sintilimab (Tyvyt, Innovent Biologics; initial dose, 200 mg every 3 weeks) was intravenously injected. According to adverse events (AEs), the dosage of lenvatinib and PD‐1 inhibitor could be halved or continued to be used after the symptoms were relieved after intermittent discontinuation.

### 2.3 Follow‐up and outcome assessment

All patients stopped follow‐up in cases of death or loss to follow‐up. According to the criteria of the National Cancer Institute for AEs, the patients were classified into grades I–IV.^47^ AEs were recorded, and blood routine, liver function, and AFP and cytokines levels were reexamined regularly during follow‐up. In addition, the patients had at least one measurable lesion at baseline treatment, and the best tumor response was estimated through contrast‐enhanced CT or MRI according to the mRECIST every 4–6 weeks, including complete remission (CR), partial remission (PR), stable disease (SD), and progressive disease (PD). Disease control rate (DCR [CR + PR + SD]) and objective response rate (ORR [CR + PR]) were also assessed. Moreover, the PFS and OS were calculated.

### 2.4 Statistical analyses

The Statistical Package for the Social Sciences version 22.0 (IBM Corp.) and GraphPad version 9.0 (GraphPad Software) were used for statistical analyses and drawing, respectively. Pearson's chi‐squared test, independent *t*‐test, and paired *t*‐test were used to analyze clinical data. Survival curves were obtained using the Kaplan–Meier method. A Cox regression model was used to determine the independent prognostic factors for survival. Statistical significance was set at *p* < 0.05.

## 3 RESULTS

### 3.1 Patient characteristics

**FIGURE 1:**
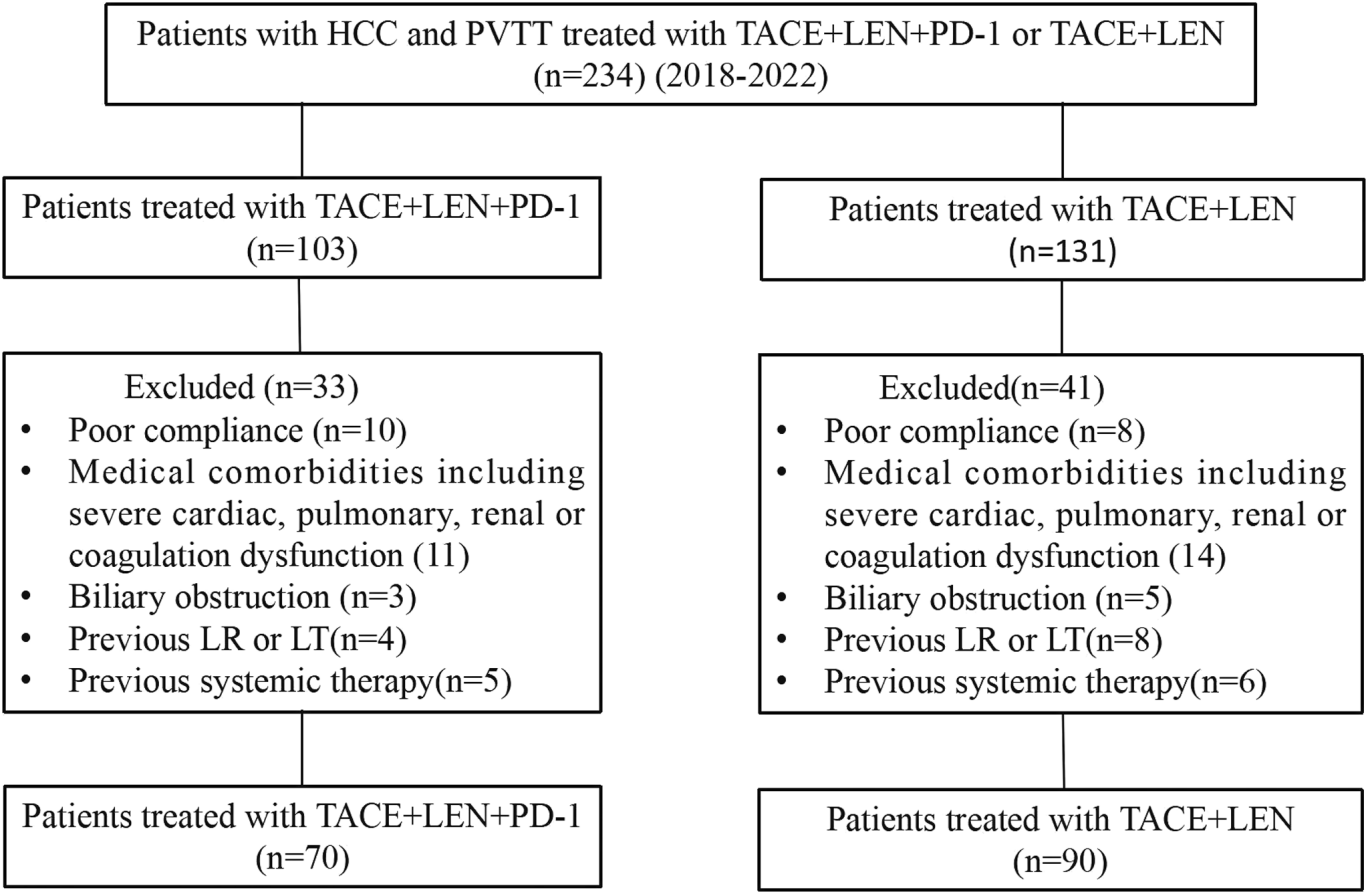
Flow diagram showed selection criteria. HCC, hepatocellular carcinoma; TACE+LEN + PD‐1, transarterial chemoembolization combined with lenvatinib plus PD‐1 inhibitor; TACE+LEN, transarterial chemoembolization combined with lenvatinib; LR, liver resection; LT, liver transplantation.

In total, 234 patients were enrolled, 160 (70 [43.75%], TACE+LEN + PD‐1 group; 90 [56.25%], TACE+LEN group) of whom met the inclusion criteria (Figure 1). The baseline characteristics of the patients in the two groups were comparable (Table 1). The two groups were similar in terms of sex, age, ECOG‐PS score, Child–Pugh grade, albumin‐bilirubin (ALBI) score, modified ALBI grade, BCLC stage C, AFP level, viral hepatitis, PVTT type, number of tumors, hepatic vein invasion, and extrahepatic metastasis. All patients were categorized into Child–Pugh A/B and BCLC C stages. Patients with PVTT VP2‐4 were included, approximately 50% of whom belonged to VP3. Viral hepatitis B accounted for almost all patients, with a minority being viral hepatitis C. For the size and number, the majority of patients with HCC had >5‐cm‐diameter tumor, and >50% patients had more than three intrahepatic tumors. Less than 15% of patients with HCC were diagnosed with extrahepatic spread. The number of TACE treatments performed in the two groups is presented in Table 1. In the TACE+LEN + PD‐1 and TACE+LEN groups, 51 (72.86%) and 76 (84.45%) patients received subsequent treatment, such as regorafenib, PD‐1 inhibitor (for patients treated with TACE+LEN), ablation, radiotherapy (including iodine‐125 seed brachytherapy), and hepatic arterial infusion chemotherapy, respectively, after disease progression. Nine (12.86%) patients in the TACE+LEN + PD‐1 group underwent curative surgical resection because of downstaging, and four patients experienced pathologic complete responses. Four (4.45%) patients in the TACE+LEN group underwent curative surgery, and none of them experienced a pathologic complete response. The remaining patients in both groups received best supportive care.

**TABLE 1:** Baseline demographic and clinical characteristics of patients enrolled in study.

Characteristics	Group		*p*
	TACE+LEN + PD‐1 (*n* = 70)	TACE+LEN (*n* = 90)	
Sex (*n*/%)			
Male	59 (84.29)	77 (85.56)	0.827
Female	11 (15.71)	13 (14.44)	
Age (median ± SD)			
Years	53.6 ± 15.1	52.3 ± 14.8	0.316
ECOG‐PS (*n*/%)			
0	17 (24.29)	28 (31.11)	0.379
1	53 (75.71)	62 (68.89)	
Child‐Pugh (*n*/%)			
A	46 (65.71%)	61 (67.78%)	0.866
B	24 (34.29%)	29 (32.22%)	
ALBI score			
—	−2.28 ± 0.54	−2.33 ± 0.47	0.750
mALBI grade			
1	39 (55.71%)	53 (58.89%)	0.929
2a	22 (31.43%)	24 (26.67%)	
2b	7 (10.00%)	10 (11.11%)	
3	2 (2.86%)	3 (3.33%)	
BCLC (*n*/%)			
C	70 (100.00)	90 (100.00)	—
Viral hepatitis (*n*/%)			
HBV	63 (90.00)	78 (86.67)	0.800
HCV	5 (7.14)	9 (10.00)	
None	2 (2.86)	3 (3.33)	
AFP (*n*/%)			
AFP ≤400 ng/mL	21 (30.00)	27 (30.00)	1.000
AFP >400 ng/mL	49 (70.00)	63 (70.00)	
Number of tumors (*n*/%)			
>3	34 (48.57)	49 (54.44)	0.524
≤3	36 (51.43)	41 (45.56)	
Type of PVTT (*n*/%)			
VP 2	18 (25.71)	22 (24.44)	0.880
VP 3	37 (52.86)	51 (56.67)	
VP 4	15 (21.43)	17 (18.89)	
Largest diameter of liver tumor (*n*/%)			
>5 cm	59 (84.29)	77 (85.56)	0.497
≤5 cm	11 (15.71)	13 (14.44)	
Hepatic vein invasion (*n*/%)			
Yes	22 (31.43)	29 (32.22)	1.000
No	48 (68.57)	61 (67.78)	
Extrahepatic metastasis (*n*/%)			
Yes	10 (14.29)	12 (13.33)	0.520
No	60 (85.71)	78 (86.67)	
TACE times			
—	3.76 ± 0.64	4.09 ± 0.52	0.401
Interval time between TACE			
Days	81.35 ± 27.55	63.72 ± 25.49	0.013
TACE technique			
cTACE	43 (61.43)	49 (54.44)	0.375
DEB‐TACE	27 (38.57)	41 (45.56)	

*Note*: Results are presented as *n* (%).

Abbreviations: AFP, alpha‐fetoprotein; ALBI, albumin‐bilirubin; BCLC, Barcelona Clinic Liver Cancer; cTACE, conventional transarterial chemoembolization; DEB‐TACE, drug‐eluting beads transarterial chemoembolization; ECOG PS, Eastern Cooperative Oncology Group Performance Status; HBV, hepatitis B virus; HCV, hepatitis C virus; mALBI, modified ALBI; PVTT, portal vein tumor thrombus; SD, standard deviation; TACE+LEN + PD‐1, transarterial chemoembolization combined with lenvatinib plus PD‐1 inhibitor; TACE+LEN, transarterial chemoembolization combined with lenvatinib.

### 3.2 Efficacy outcomes

In the follow‐up until May 2022, 56 (77.14%) patients in the TACE+LEN + PD‐1 group and 71 (78.89%) patients in the TACE+LEN group died. The best tumor response in 160 patients in the two groups was evaluated based on the mRECIST criteria (Table 2). The ORRs were 38.57% in the TACE+LEN + PD‐1 group and 24.45% in the TACE+LEN group. The DCRs were 80.00% in the TACE+LEN + PD‐1 group and 56.67% in the TACE+LEN group. The ORR and DCR in the TACE+LEN + PD‐1 group were significantly higher than those in the TACE+LEN group (*p* = 0.025), suggesting that TACE+LEN + PD‐1 has a better antitumor effect than TACE+LEN.

**TABLE 2:** Best tumor response in patients to TACE+LEN + PD‐1 group and TACE+LEN group.

Tumor response	TACE+LEN + PD‐1 (*n* = 70)	TACE+LEN (*n* = 90)	*χ* ^*2*^	*p*
CR	7 (10.00%)	5 (5.56%)	12.809	0.025
PR	20 (28.57%)	17 (18.89%)		
SD	29 (41.43%)	29 (32.22%)		
PD	14 (20.00%)	39 (43.33%)		
ORR (CR + PR)	27 (38.57%)	22 (24.45%)		
DCR (CR + PR + SD)	56 (80.00%)	51 (56.67%)		

*Note*: Results are presented as *n* (%).

Abbreviations: CR, complete response; DCR (CR + PR + SD), disease control rate; ORR(CR + PR), objective response rate; PD, progressive disease; PR, partial response; SD, stable disease; TACE+LEN + PD‐1, transarterial chemoembolization combined with lenvatinib plus PD‐1 inhibitor; TACE+LEN, transarterial chemoembolization combined with lenvatinib.

**FIGURE 2:**
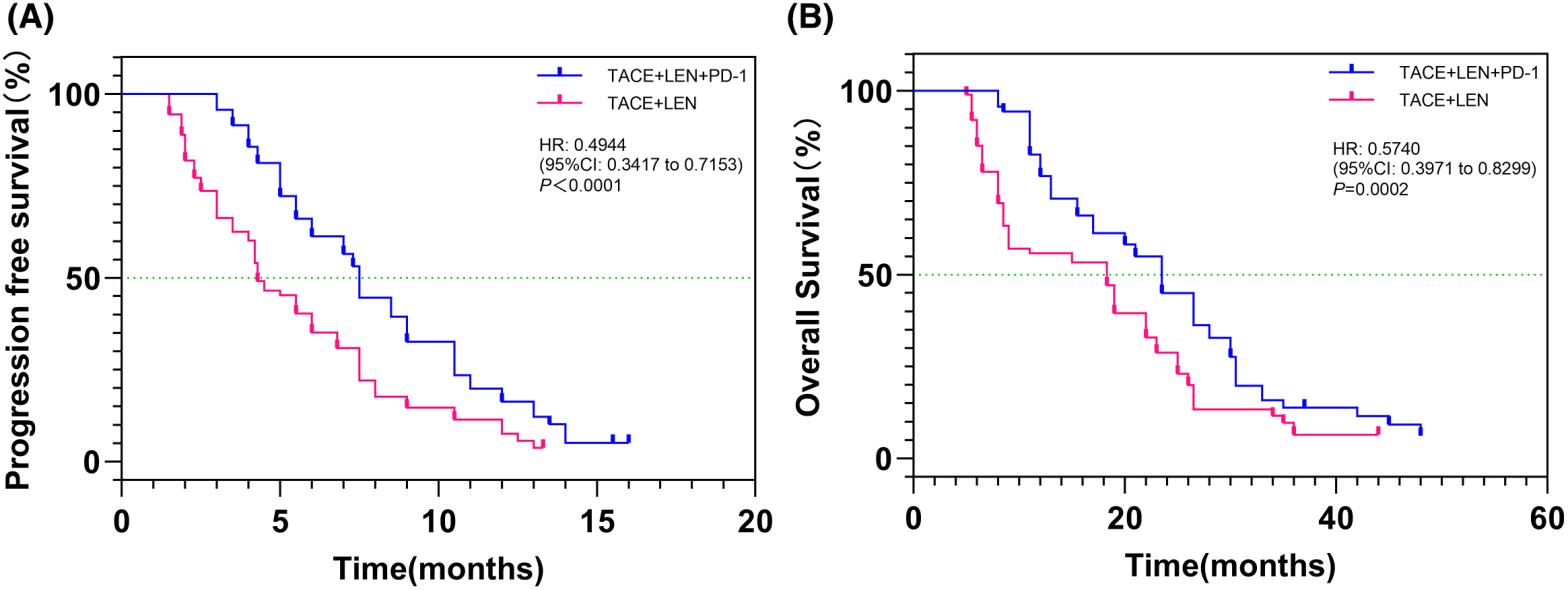
Kaplan–Meier curves of (A) progression‐free survival and (B) overall survival in the TACE+LEN + PD‐1 group and TACE+LEN group. TACE+LEN + PD‐1, transarterial chemoembolization combined with lenvatinib plus PD‐1 inhibitor; TACE+LEN, transarterial chemoembolization combined with lenvatinib; HR, hazard ratio; CI, confidence interval.

**FIGURE 3:**
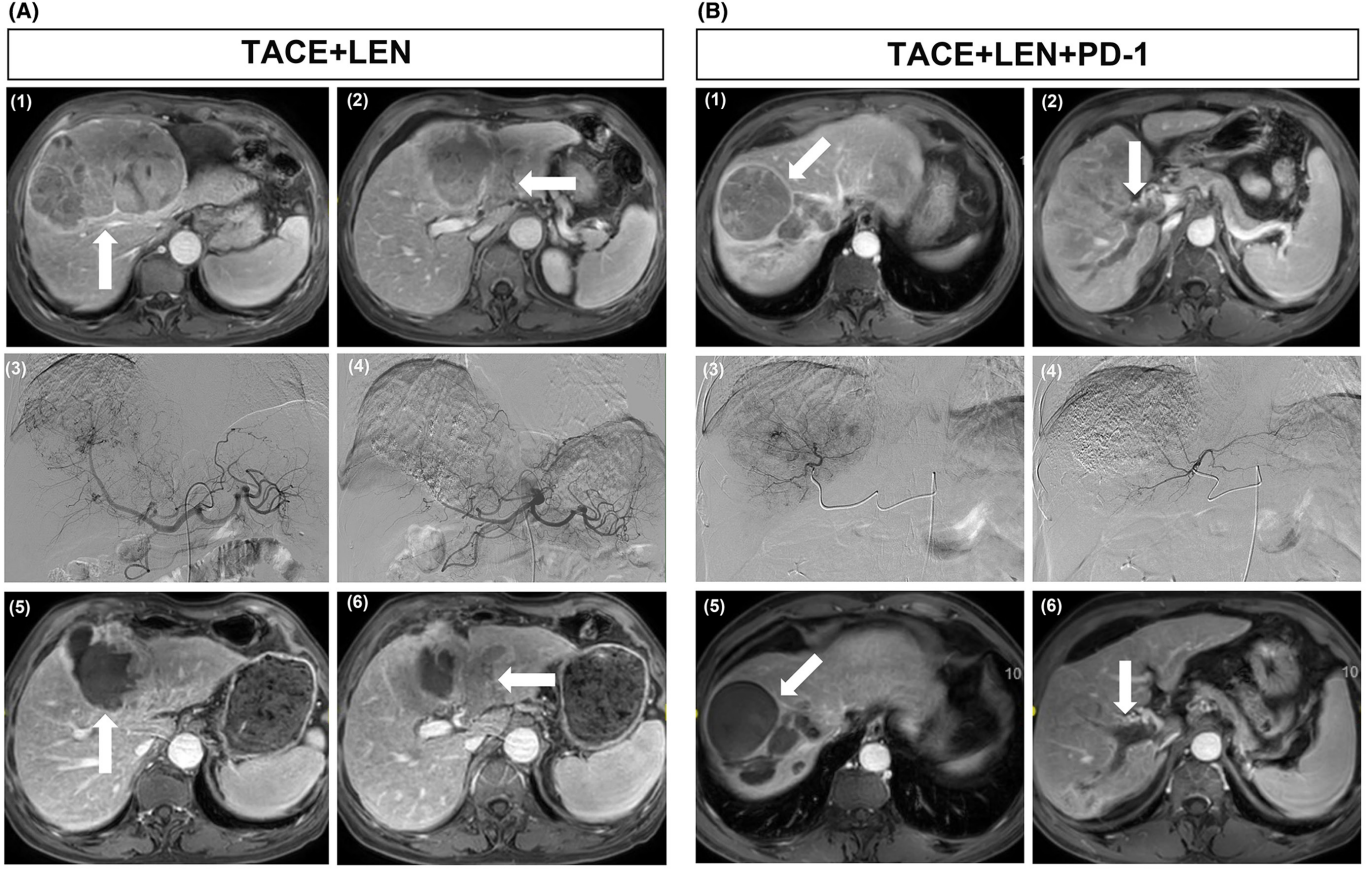
(A) MRI image of the liver obtained from a 49‐year‐old male patient with a history of hepatitis B for 20 years who had been treated with TACE+LEN. (1) (2) Contrast‐enhanced MRI showed the presence of HCC with the tumor thrombus type of VP3 in the left portal vein (arrow shown). (3) (4) Digital subtraction angiography (DSA) showed obvious staining of HCC when undergoing TACE. (5) (6) Three months after TACE+LEN treatment, contrast‐enhanced MRI showed although the tumor was mostly controlled, there was still blood supply in the PVTT (arrow shown). (B) MRI image of the liver obtained from a 55‐year‐old male patient with a history of hepatitis B for 30 years who had been treated with TACE+LEN + PD‐1. (1) (2) Contrast‐enhanced MRI showed the presence of HCC with the tumor thrombus type of VP3 in the right portal vein (arrow shown). (3) (4) DSA showed obvious staining of HCC when undergoing TACE. (5) (6) Three months after TACE+LEN + PD‐1 treatment, contrast‐enhanced MRI showed HCC and PVTT were necrotic, and no obvious blood supply was found in the lesions (arrow shown).

The TACE+LEN + PD‐1 group had significantly more prolonged mPFS period compared with the TACE+LEN group (7.5 vs 4.3 months; HR = 0.4944; 95% CI, 0.3417–0.7153; *p* < 0.0001; Figure 2A). The median OS periods were 23.5 months for the TACE+LEN + PD‐1 group and 18.3 months for the TACE+LEN group (HR = 0.5740; 95% CI, 0.3971–0.8299; *p* = 0.0002; Figure 2B). The tumor was well controlled with PVTT necrosis, and there was no distant metastasis in the TACE+LEN + PD‐1 group. However, in the TACE+LEN group, although the tumor was mostly controlled, there was still blood supply in the PVTT (Figure 3).

### 3.3 Biochemical blood indices

Biochemical blood indices were compared between the two groups 4–6 weeks after the initial treatment. The detection results of tumor markers, liver function, routine blood tests, and tumor immune‐related cytokines before treatment in the two groups were similar (*p* > 0.05, Table 3). After treatment, in terms of liver function, the levels of aspartate aminotransferase, alanine aminotransferase (ALT), and cholinesterase (CHE), as well as prothrombin time and AFP level as indicators of coagulation function and tumor marker, respectively, in the two groups of patients were better compared with those before treatment (*p* < 0.05, Table 4). In contrast, improvements in the levels of ALT, CHE, AFP, and international normalized ratio were better in the TACE+LEN + PD‐1 group than in the TACE+LEN group after treatment (*p* < 0.05), indicating that TACE+LEN + PD‐1 treatment has promising efficacy associated with antitumor effects and improvement of liver and coagulation functions.

**TABLE 3:** Comparison of biochemical indices before and after treatment between two groups.

Indicators	Before treatment				After treatment			
	TACE + LEN + PD‐1 (*n* = 70)	TACE + LEN (*n* = 90)	*t*	*p*	TACE + LEN + PD‐1 (*n* = 70)	TACE + LEN (*n* = 90)	*t*	*p*
TBIL (μmol/L)	24.46 ± 3.79	27.03 ± 4.85	13.089	0.157	19.32 ± 3.23	18.17 ± 3.11	19.473	0.610
ALB (g/L)	36.13 ± 3.61	37.15 ± 2.87	−21.065	0.091	38.25 ± 4.49	37.91 ± 3.05	−2.817	0.401
AST (U/L)	43.08 ± 13.65	46.25 ± 14.61	−6.562	0.137	41.83 ± 11.14	40.72 ± 12.81	17.925	0.319
ALT (U/L)	41.49 ± 12.97	48.37 ± 13.56	9.353	0.734	37.71 ± 10.35	43.35 ± 10.77	−29.261	0.011
CHE (U/L)	4287.39 ± 793.61	4296.85 ± 730.92	8.921	0.097	4845.08 ± 869.73	4787.63 ± 925.33	4.068	0.031
PT (s)	12.01 ± 1.89	12.62 ± 2.13	5.539	0.087	11.57 ± 2.03	12.05 ± 1.97	−5.381	0.517
INR	1.12 ± 0.20	1.13 ± 0.17	19.516	0.534	1.01 ± 0.15	1.15 ± 0.19	−0.109	0.014
AFP (μg/L)	869.90 ± 245.95	907.34 ± 259.83	5.615	0.329	271.87 ± 73.05	375.46 ± 79.80	−6.816	0.000
TNF‐α (pg/mL)	46.37 ± 12.09	41.91 ± 10.78	10.219	0.272	39.45 ± 10.23	43.57 ± 11.06	−11.218	0.098
IL‐1β (pg/mL)	31.55 ± 9.35	35.87 ± 9.79	−15.761	0.431	17.01 ± 4.08	30.29 ± 6.77	−20.860	0.012
IL‐2 (pg/mL)	35.28 ± 11.45	39.34 ± 11.50	−22.096	0.107	31.39 ± 8.77	36.55 ± 9.35	−31.203	0.087
IL‐6 (pg/mL)	59.75 ± 13.26	52.39 ± 11.91	32.780	0.357	35.26 ± 8.03	48.35 ± 10.91	−17.306	0.003
IL‐17 (pg/mL)	40.33 ± 8.73	43.65 ± 9.79	−9.751	0.232	17.21 ± 3.29	44.12 ± 11.05	−13.299	0.006
CD4+/CD8+	0.84 ± 0.09	0.91 ± 0.12	−13.097	0.811	1.66 ± 0.23	1.01 ± 0.18	28.779	0.000
IFN‐α (pg/mL)	21.87 ± 5.31	18.92 ± 5.77	18.919	0.328	8.01 ± 2.87	15.06 ± 3.23	−15.356	0.000
IFN‐γ (pg/mL)	25.67 ± 6.11	24.83 ± 7.04	10.093	0.109	23.65 ± 5.79	21.99 ± 5.82	13.097	0.673
VEGF (pg/mL)	238.56 ± 19.77	229.79 ± 17.80	16.552	0.379	141.22 ± 15.39	168.79 ± 14.17	−25.771	0.020

*Note*: Results are presented as mean ± SD.

Abbreviations: AFP, alpha‐fetoprotein; ALB, albumin; ALT, alanine transaminase; AST, aspartate aminotransferase; CHE, cholinesterase; INR, international normalized ratio; PT, prothrombin time; TACE+LEN + PD‐1, transarterial chemoembolization combined with lenvatinib plus PD‐1 inhibitor; TACE+LEN, transarterial chemoembolization combined with lenvatinib; TBIL, total bilirubin.

**TABLE 4:** Comparison of biochemical indices in two groups before and after treatment.

Indicators	TACE+LEN + PD‐1 (*n* = 70)				TACE + LEN (*n* = 90)			
	Before treatment	After treatment	*t*	*p*	Before treatment	After treatment	*t*	*p*
TBIL (μmol/L)	24.46 ± 3.79	19.32 ± 3.23	−30.651	0.132	27.03 ± 4.85	18.17 ± 3.11	−36.297	0.307
ALB (g/L)	36.13 ± 3.61	38.25 ± 4.49	−49.857	0.203	37.15 ± 2.87	37.91 ± 3.05	−11.279	0.906
AST (U/L)	43.08 ± 13.65	41.83 ± 11.14	13.218	0.002	46.25 ± 14.61	40.72 ± 12.81	30.187	0.039
ALT (U/L)	41.49 ± 12.97	37.71 ± 10.35	75.052	0.011	48.37 ± 13.56	43.35 ± 10.77	15.715	0.022
CHE (U/L)	4287.39 ± 793.61	4845.08 ± 869.73	−39.231	0.002	4296.85 ± 730.92	4787.63 ± 925.33	−38.914	0.003
PT (s)	12.01 ± 1.89	11.57 ± 2.03	75.109	0.013	12.62 ± 2.13	12.05 ± 1.97	21.330	0.026
INR	1.12 ± 0.20	1.01 ± 0.15	6.557	0.008	1.13 ± 0.17	1.15 ± 0.19	−23.201	0.177
AFP (μg/L)	869.90 ± 245.95	271.87 ± 73.05	10.258	0.000	907.34 ± 259.83	375.46 ± 79.80	11.203	0.000
TNF‐α (pg/mL)	46.37 ± 12.09	39.45 ± 10.23	12.309	0.712	41.91 ± 10.78	43.57 ± 11.06	−9.733	0.871
IL‐1β (pg/mL)	31.55 ± 9.35	17.01 ± 4.08	30.13	0.035	35.87 ± 9.79	30.29 ± 6.77	19.76	0.231
IL‐2 (pg/mL)	35.28 ± 11.45	31.39 ± 8.77	12.92	0.082	39.34 ± 11.50	36.55 ± 9.35	41.23	0.779
IL‐6 (pg/mL)	59.75 ± 13.26	35.26 ± 8.03	19.37	0.010	52.39 ± 11.91	48.35 ± 10.91	21.58	0.136
IL‐17 (pg/mL)	40.33 ± 8.73	17.21 ± 3.29	15.33	0.000	43.65 ± 9.79	44.12 ± 11.05	−10.26	0.373
CD4+/CD8+	0.84 ± 0.09	1.66 ± 0.23	−17.99	0.013	0.91 ± 0.12	1.05 ± 0.18	−13.50	0.328
IFN‐α (pg/mL)	21.87 ± 5.31	8.01 ± 2.87	19.83	0.000	18.92 ± 5.77	15.06 ± 3.23	20.76	0.437
IFN‐γ (pg/mL)	25.67 ± 6.11	23.65 ± 5.79	13.87	0.451	24.83 ± 7.04	21.99 ± 5.82	13.90	0.210
VEGF (pg/mL)	238.56 ± 19.77	141.22 ± 15.39	47.91	0.001	229.79 ± 17.80	168.79 ± 14.17	39.06	0.013

*Note*: Results are presented as mean ± SD.

Abbreviations: AFP, alpha‐fetoprotein; ALB, albumin; ALT, alanine transaminase; AST, aspartate aminotransferase; CHE, cholinesterase; INR, international normalized ratio; PT, prothrombin time; TACE+LEN + PD‐1, transarterial chemoembolization combined with lenvatinib plus PD‐1 inhibitor; TACE+LEN, transarterial chemoembolization combined with lenvatinib; TBIL, total bilirubin.

Notably, tumor immune‐related cytokines, including tumor necrosis factor (TNF)‐α, IL‐1β, IL‐2, IL‐6, IL‐17, CD4+/CD8+, IFN‐α, and IFN‐γ, were determined.

The levels of IL‐1β, IL‐6, IL‐17, and IFN‐α decreased after treatment in the TACE+LEN + PD‐1 group, and treatment with PD‐1 inhibitor in the TACE+LEN + PD‐1 group was superior to that in the TACE+LEN group in reducing the level of these tumor‐related factors, which may be attributed to the blocking effect of the PD‐1 inhibitor immune pathway (Tables 3 and 4). Moreover, VEGF plays a vital role in the occurrence and development of tumors, and treatment with the VEGF inhibitor lenvatinib reduced the level of VEGF in the serum, which was reflected in the treatment of the two groups, in which the TACE+LEN + PD‐1 group was more significant in reducing the level of VEGF than the TACE+LEN group.

### 3.4 Prognostic factor analysis for OS and progression‐free survival

An additional Cox regression analysis was performed for OS and PFS in both groups (Table 5). Based on the results of univariate and multivariate analyses, Child–Pugh grade (B/A; HR = 1.793, 95% CI, 1.105–2.511; *p* = 0.008), PVTT classification (VP4/VP3/VP2; HR = 1.921; 95% CI, 1.497–2.315; *p =* 0.002); treatment option (TACE+LEN/TACE+LEN + PD‐1; HR = 2.305; 95% CI, 1.509–2.818; *p* = 0.003), and IL‐6 (HR = 1.797, 95% CI, 0.902–2.356; *p =* 0.001), IL‐17 (HR = 1.314; 95% CI, 0.936–1.820; *p* = 0.014), IFN‐α (HR = 1.530; 95% CI, 0.987–2.018; *p* = 0.011), and VEGF (HR = 1.612; 95% CI, 1.116–2.031; *p* = 0.004) levels were the independent prognostic factors for OS. In addition, PVTT classification (HR = 1.911; 95% CI, 1.377–2.572; *p* = 0.025), treatment option (HR = 2.091; 95% CI, 1.439–2.752; *p* = 0.005), and IL‐6 (HR = 1.446; 95% CI, 0.869–1.799; *p* = 0.014) and IFN‐α (HR = 1.327; 95% CI, 0.931–1.967; *p* = 0.035) levels were the independent factors for PFS.

**TABLE 5:** Analyses of prognostic factors for survival.

Factors	Overall survival				Progression‐free survival			
	Univariate analysis		Multivariate analysis		Univariate analysis		Multivariate analysis	
	HR (95% CI)	*p*	HR (95% CI)	*p*	HR (95% CI)	*p*	HR (95% CI)	*p*
Sex								
Female/Male	0.910 (0.684–1.223)	0.415			0.803 (0.528–1.210)	0.197		
Age (years)								
<60/≥60	0.981 (0.809–1.117)	0.147			0.859 (0.710–1.079)	0.308		
ECOG‐PS								
1/0	1.134 (0.903–1.217)	0.366			1.055 (0.891–1.269)	0.401		
Child‐Pugh grade								
B/A	2.465 (1.363–3.901)	<0.001	1.793 (1.105–2.511)	0.008	2.125 (1.393–2.806)	0.003	1.190 (0.681–1.597)	0.102
Viral hepatitis								
HBV/HCV/Negative	1.010 (0.835–1.338)	0.117			0.873 (0.617–1.216)	0.140		
AFP (μg/L)								
≤400/>400	0.915 (0.710–1.395)	0.326			1.013 (0.702–1.252)	0.193		
Number of tumors								
≤3/>3	0.893 (0.417–1.280)	0.619			0.791 (0.479–1.215)	0.518		
PVTT classification								
VP4/VP3/VP2	2.510 (1.699–3.108)	<0.001	1.921 (1.497–2.315)	0.002	2.033 (1.511–2.836)	0.001	1.911 (1.377–2.572)	0.025
Tumor size (cm)								
≤5/>5	0.729 (0.501–0.906)	0.026	0.981 (0.743–1.179)	0.655	0.816 (0.588–1.117)	0.076		
Hepatic vein invasion								
Yes/No	1.102 (0.835–1.302)	0.060			1.703 (0.854–2.015)	0.029	1.041 (0.663–1.379)	0.153
Extrahepatic metastasis								
Yes/No	1.326 (0.879–2.055)	0.001	0.977 (0.894–1.130)	0.104	1.103 (0.809–1.598)	0.106		
Treatment option								
TACE+LEN/TACE+LEN + PD‐1	2.037 (1.498–2.671)	0.015	2.305 (1.509–2.818)	0.003	1.834 (1.293–2.551)	0.009	2.091 (1.439–2.752)	0.005
TNF‐α (pg/mL)	0.915 (0.833–1.106)	0.031	0.989 (0.903–1.021)	0.213	1.012 (0.821–1.153)	0.219		
IL‐1β (pg/mL)	1.328 (0.805–1.781)	0.013	0.969 (0.457–1.198)	0.411	1.201 (0.803–1.533)	0.287		
IL‐2 (pg/mL)	0.904 (0.423–1.277)	0.551			0.914 (0.408–1.231)	0.680		
IL‐6 (pg/mL)	2.601 (1.811–3.597)	<0.001	1.797 (0.902–2.356)	0.001	2.414 (1.802–3.435)	0.002	1.446 (0.869–1.799)	0.014
IL‐17 (pg/mL)	1.598 (1.102–1.979)	0.005	1.314 (0.936–1.820)	0.014	1.002 (0.747–1.215)	0.094		
CD4+/CD8+	1.207 (0.713–1.621)	0.019	0.973 (0.338–1.310)	0.478	1.236 (0.825–1.768)	0.003	0.989 (0.435–1.504)	0.198
IFN‐α (pg/mL)	1.902 (1.105–2.223)	0.005	1.530 (0.987–2.018)	0.011	1.917 (1.205–2.451)	0.001	1.327 (0.931–1.967)	0.035
IFN‐γ (pg/mL)	0.913 (0.356–1.127)	0.302			0.879 (0.351–1.203)	0.177		
VEGF (pg/mL)	1.979 (1.375–2.317)	<0.001	1.612 (1.116–2.031)	0.004	1.625 (1.107–2.122)	0.014	0.916 (0.620–1.306)	0.112

Abbreviations: AFP, alpha‐fetoprotein; CI, confidence interval; ECOG PS, Eastern Cooperative Oncology Group Performance Status; HBV, hepatitis B virus; HCV, hepatitis C virus; HR, hazard ratio; PVTT, portal vein tumor thrombus.

### 3.5 Subgroup analyses

**FIGURE 4:**
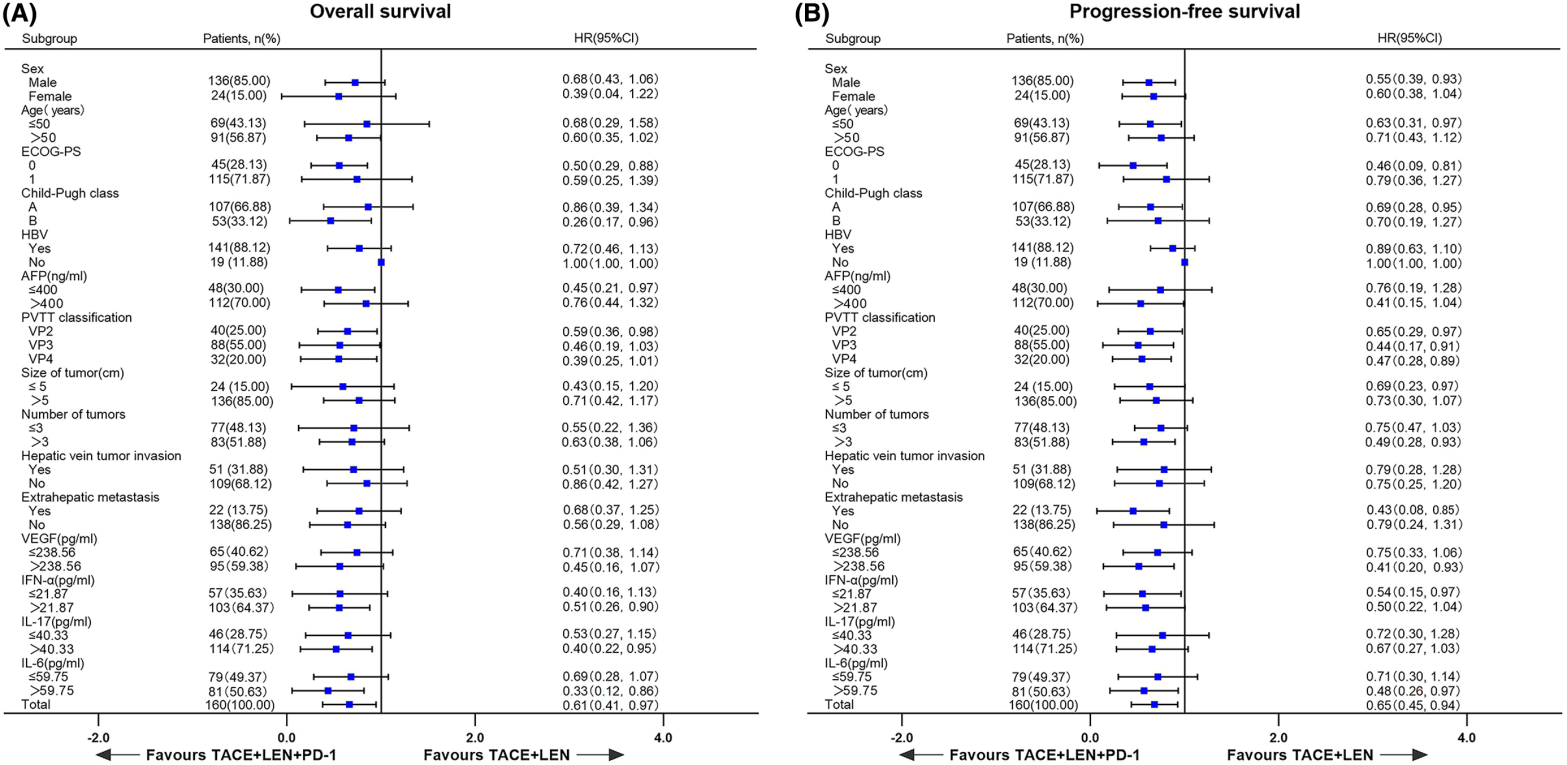
Forest plots of (A) overall survival and (B) progression‐free survival in subgroups of patients treated with TACE+LEN + PD‐1 and TACE+LEN.

Forest plots of the subgroup analyses for OS and PFS in the TACE+LEN + PD‐1 and TACE+LEN groups are shown in Figure 4. Subgroup analysis showed that TACE+LEN + PD‐1 was associated with better OS and PFS than TACE+LEN across most patient subgroups, especially for Child–Pugh grade (A/B), PVTT classification (VP2/VP3/VP4), and tumor immune‐related cytokine levels, which were associated with prognosis.

**FIGURE 5:**
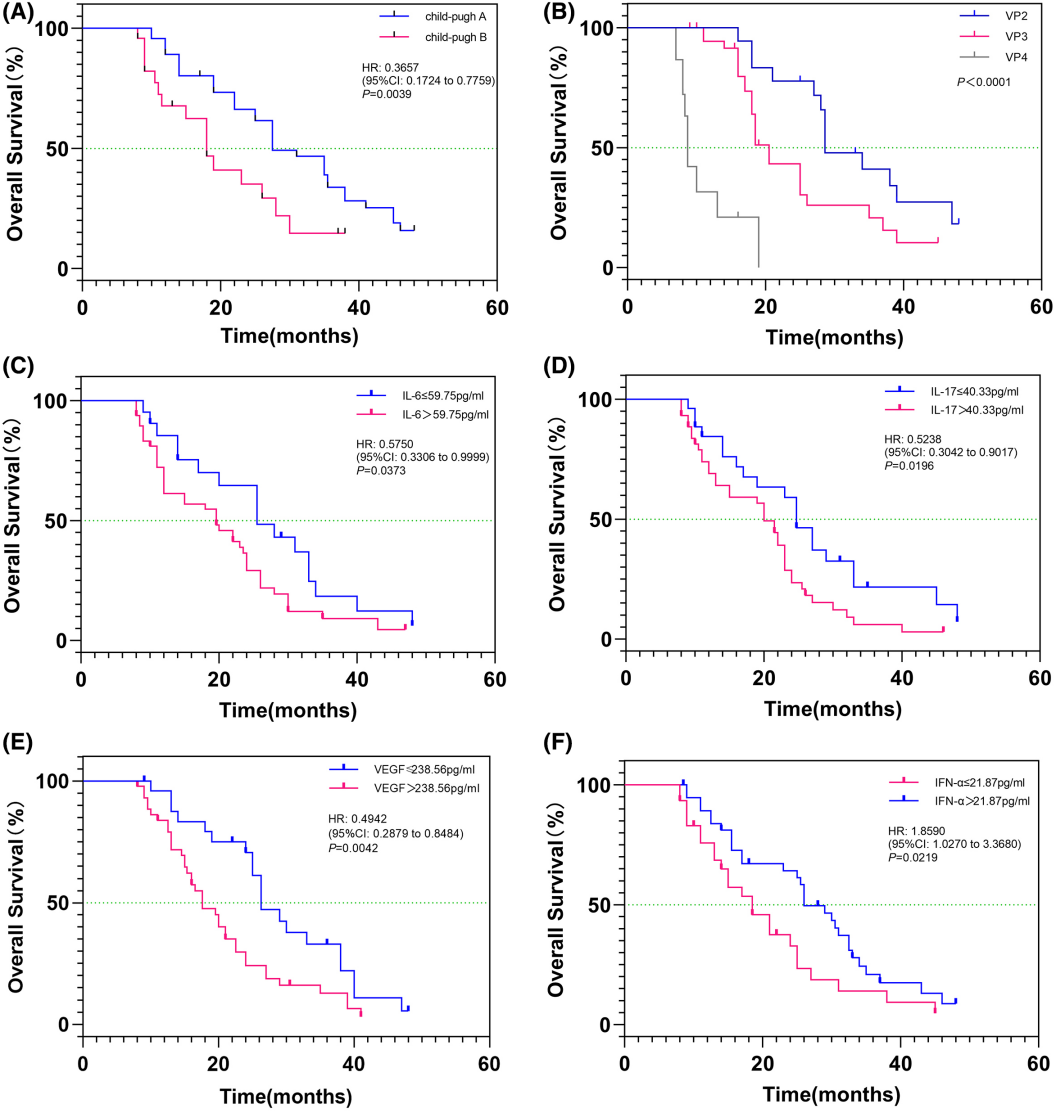
Survival analyses of independent prognostic factors in TACE+LEN + PD‐1 group. (A) Child‐Pugh grade; (B) PVTT classification; (C) IL‐6; (D) IL‐17; (E) VEGF; (F) IFN‐α. TACE+LEN + PD‐1, transarterial chemoembolization combined with lenvatinib plus PD‐1 inhibitor; PVTT, portal vein tumor thrombus; IL‐6, interleukin‐6; IL‐17, interleukin‐17; VEGF, vascular endothelial growth factor; IFN‐α, interferon‐α.

Furthermore, based on the results of the superior subgroups and independent prognostic factors for OS, stratified analysis was performed (Figure 5). The effects of liver function and tumor‐related factors on OS were analyzed (Figure 5a,b). In the TACE+LEN + PD‐1 group, the patients were classified as Child–Pugh A/B and PVTT VP2/VP3/VP4. The mean OS periods were 27.5 months for patients with Child–Pugh A and 18.0 months for patients with Child–Pugh B (HR = 0.3657; 95% CI, 0.1724–0.7759; *p* = 0.0039; Figure 5A). The mean OS periods in the VP2 and VP3 of PVTT type were longer than those in VP4 (28.6 months in VP2 and 20.5 months in VP3 vs. 8.7 months in VP4, *p* < 0.0001, Figure 5B). Patients with Child–Pugh grade A and PVTT VP2/VP3 had more significant clinical outcomes related to OS after TACE+LEN + PD‐1 treatment.

Next, stratified analysis of tumor immune‐related cytokines affecting survival and prognosis was performed. The mean values of IL‐6, IL‐17, IFN‐α, and VEGF before treatment in the TACE+LEN + PD‐1 group were used as the boundaries for stratified analysis. The mean values of IL‐6, IL‐17, IFN‐α, and VEGF were 59.75, 40.33, 21.87, and 238.56 pg/mL, respectively. Subgroup analyses revealed that the mean OS period in patients with low IL‐6 level (≤59.75 pg/mL) was significantly more prolonged than that in patients with high IL‐6 level (>59.75 pg/mL) (25.5 vs. 19.6 months; HR = 0.5750; 95% CI, 0.3306–0.9999; *p* = 0.0373; Figure 5C). Moreover, the mean OS period in patients with low IL‐17 level was significantly more prolonged than that in patients with high IL‐17 level (24.7 months in patients with IL‐17 ≤ 40.33 pg/mL vs. 20.0 months in patients with IL‐17 > 40.33 pg/mL; HR = 0.5238; 95% CI, 0.3042–0.9017; *p* = 0.0196; Figure 5D). Furthermore, the mean OS period in patients with low VEGF level was significantly more prolonged than that in patients with high VEGF level (26.3 months in patients with VEGF ≤238.56 pg/mL vs. 17.6 months in patients with VEGF >238.56 pg/mL; HR = 0.4942; 95% CI, 0.2879–0.8484; *p* = 0.0042; Figure 5E). In contrast, the mean OS periods were 18.5 months in patients with low IFN‐α level (≤21.87 pg/mL) and 26.0 months in patients with high IFN‐α level (>21.87 pg/mL) (HR = 1.8590; 95% CI, 1.0270–3.3680; *p* = 0.0219; Figure 5F).

### 3.6 Safety outcomes

All reported AEs were evaluated, and no treatment‐related deaths occurred in either group. Details of the AEs are summarized in Table 6. The most frequent AEs in the TACE+LEN + PD‐1 group were hand‐foot syndrome (20.00%), hypertension (25.71%), anorexia and nausea (27.14%), rash (24.29%), fatigue (28.57%), and hyperbilirubinemia (21.34%). The most important grade 3–4 AEs were hand‐foot syndrome (8.57%), hyperbilirubinemia (10.00%), and thyroid dysfunction (8.57%). Similarly, in the TACE+LEN group, the most frequent AEs were hand‐foot syndrome (18.89%), hypertension (27.78%), anorexia and nausea (30.00%), rash (16.67%), fatigue (31.11%), and hyperbilirubinemia (21.11%). More grade 3–4 toxicities were observed in hand‐foot syndrome (5.56%) and fatigue (6.67%). The frequency and severity of AEs were similar between the two groups, and the AEs in the two groups were alleviated after drug reduction and symptomatic treatment. Owing to serious AEs, 31 (44.29%) patients in the TACE+LEN + PD‐1 group and 33 (36.67%) patients in the TACE+LEN group received dose reduction. In the TACE+LEN + PD‐1 group, the mean lenvatinib dose intensities were 7.1 mg in the low‐dose group (8 mg/day) and 9.8 mg in the high‐dose group (12 mg/day). The corresponding dose intensities in the TACE+LEN group were 7.5 and 10.2 mg, respectively. The mean PD‐1 inhibitor dose intensity was 163.8 mg in the TACE+LEN + PD‐1 group.

**TABLE 6:** Treatment‐related adverse events in the two groups.

Adverse events	TACE+LEN + PD‐1 (*n* = 70)		TACE+LEN (*n* = 90)		*χ* ^*2*^	*p*
	Any grade	Grade 3–4	Any grade	Grade 3–4		
Diarrhea	11 (15.71)	3 (4.29)	13 (14.44)	4 (4.44)	0.019	0.889
Hand‐foot syndrome	14 (20.00)	6 (8.57)	17 (18.89)	5 (5.56)	0.287	0.592
Hypertension	18 (25.71)	3 (4.29)	25 (27.78)	3 (3.33)	0.142	0.706
Fatigue	20 (28.57)	5 (7.14)	28 (31.11)	6 (6.67)	0.053	0.819
Anorexia and nausea	19 (27.14)	2 (2.86)	27 (30.00)	3 (3.33)	0.003	0.955
Rash	17 (24.29)	3 (4.29)	15 (16.67)	2 (2.22)	0.082	0.774
Oral ulcer	14 (20.00)	5 (7.14)	16 (17.78)	4 (4.44)	0.219	0.640
Trachyphonia	7 (10.00)	1 (1.43)	9 (10.00)	2 (2.22)	0.112	0.737
Thyroid dysfunction	8 (11.43)	6 (8.57)	7 (7.78)	0 (0.00)	4.200	0.060
Hyperbilirubinemia	15 (21.43)	7 (10.00)	19 (21.11)	2 (2.22)	3.227	0.072
Proteinuria	7 (10.00)	0 (0.00)	5 (5.56)	1 (1.11)	1.264	0.261
Thrombocytopenia	12 (17.14)	3 (4.29)	14 (15.56)	5 (5.56)	0.186	0.666

*Note*: Results are presented as *n* (%).

Abbreviations: TACE+LEN + PD‐1, transarterial chemoembolization combined with lenvatinib plus PD‐1 inhibitor; TACE+LEN, transarterial chemoembolization combined with lenvatinib.

## 4 DISCUSSION

Most patients with HCC are accompanied by PVTT, which is essential for tumor staging and is characterized by poor prognosis and limited treatment choices and survival time.^7^, ^8^ Currently, despite the progress in the treatment of advanced HCC, including first‐line therapy (sorafenib, lenvatinib, and Atezo/Bev) and second‐line therapy (regorafenib, cabozantinib, pembrolizumab, and ramucirumab), the optimal treatment for HCC with PVTT remains controversial owing to poor prognosis, patient tolerance, and tumor resistance. Although the application of immune checkpoint inhibitors or TKIs alone cannot achieve significant clinical outcomes, it is satisfactory to combine immunotherapy with systemic therapy according to previous phase Ib and IMbrave150 trials.^28^, ^33^

Moreover, TACE treatment has promising clinical outcomes and acceptable safety for patients with HCC and PVTT.^48^, ^49^, ^50^ Even in patients with HCC and PVTT classification VP4, if there are compensated collateral vessels of the portal vein and fine reserve of liver function or if portal vein blood flow is regained through portal vein stent implantation, interventional therapy can prolong the survival time without increasing the risk of death caused by treatment.^51^, ^52^, ^53^ Several studies have employed TACE combined with sorafenib or lenvatinib for the treatment of unresectable HCC.^27^, ^54^, ^55^ In 2022, the LAUNCH trial found that the combination of lenvatinib and TACE as a first‐line treatment for advanced HCC could effectively prolong the survival of patients with HCC.^17^ The possible mechanism is that TACE induces hypoxia and promotes tumor angiogenesis to upregulate proangiogenic factors (VEGF and PDGF) and that the combination of sorafenib or lenvatinib treatment plays the largest antiangiogenic role by inhibiting the upregulation of proangiogenic factors following TACE in unresectable HCC. There is growing evidence that TACE plus lenvatinib presents more favorable outcomes compared with TACE plus sorafenib in patients with HCC and PVTT.^27^, ^56^, ^57^

Nonetheless, not all patients can benefit from TACE combined with lenvatinib, such as patients with extrahepatic metastasis, which may be associated with the mechanism of escape recognition by T cells. The immune evasion capacity of cancer cells may be the reason why HCC can escape from TACE plus lenvatinib and other traditional treatment strategies.^58^, ^59^, ^60^ Furthermore, the efficacy and safety of immune checkpoint inhibitors, including pembrolizumab and camrelizumab, that inhibit immune escape through the PD‐1/PD‐L1 axis have been demonstrated for advanced HCC in multiple previous trials, which seems to inhibit the consequences of tumor escape caused by traditional treatment, thus playing a complementary role.^29^, ^61^ Therefore, the triple combination treatment of TACE+LEN + PD‐1 might be a promising clinical outcome and an acceptable safety treatment strategy for advanced HCC, including PVTT. Our study demonstrated that TACE+LEN + PD‐1 was superior to TACE+LEN in mOS (23.5 vs. 18.3 months, *p* = 0.0002) and mPFS (7.5 vs. 4.3 months, *p* < 0.0001). Moreover, TACE+LEN + PD‐1 showed significantly better benefits with regard to DCR (80.00% vs. 56.67%) and ORR (38.57% vs 24.45%) than TACE+LEN in patients with HCC and PVTT (*p* = 0.025). The present study found that TACE+LEN + PD‐1 had significantly better mOS and mPFS than those reported in previous trials, including the IMbrave150 (mPFS, 6.8 months) and phase Ib trial (mOS, 22 months) trials.^28^, ^33^ Previous studies have assessed the combined TACE+LEN + PD‐1 treatment for unresectable HCC, with PFS and OS periods of 8.5–13.3 and 23.6–28.9 months, respectively, which seem to be more prolonged than those of patients treated with TACE+LEN + PD‐1 in our study.^34^, ^35^, ^62^ Notably, these studies have included a large percentage (25.0%–43.4%) of patients with BCLC stage B HCC and Child–Pugh grade A (>80%). Their clinical outcomes are expected to be better than those of patients with HCC with BCLC stage C (100%) and Child–Pugh grade A (60%) included in our study.

However, the definite mechanism of TACE+LEN + PD‐1 is uncertain. It is possible that VEGF released by TACE‐induced hypoxia is inhibited by lenvatinib, a multikinase inhibitor, by inhibiting the VEGF receptor, leading to the reprogramming of the immunosuppressive tumor microenvironment into an immunostimulatory environment and enhancing the immune regulatory activity of PD‐1 inhibitor in HCC.^29^ VEGF creates an immunosuppressive microenvironment through the following three mechanisms: (1) enhancing proliferation of regulatory T cells (Tregs) and myeloid‐derived suppressor cells (MDSCs) to release more immunosuppressive cytokines, (2) preventing T cells from infiltrating tumor tissues, and (3) reducing T‐cell proliferation and activation by suppressing the activation and maturation of dendritic cells (DCs). Therefore, VEGF inhibitors may reverse the immunosuppressive state of the tumor microenvironment and improve the immune response rate of PD‐1/PD‐L1 inhibitors through the following mechanisms: (1) inhibiting the expansion of Tregs and immunosuppressive MDSCs, (2) promoting the infiltration of T cells in tumor tissues through the normalization of tumor vasculature and activation of endotheliocytes; (3) inducing the expression of E‐selectin in endotheliocytes, thus stimulating the extravasation of lymphocytes into the tumor interstitial space; and (4) inhibiting nuclear factor‐κB to induce the maturation of DCs.^29^, ^63^, ^64^ In summary, TACE+LEN + PD‐1 may result in cooperative antitumor activity, contributing to improved clinical outcomes in patients with HCC and PVTT.

Currently, it remains uncertain which type of patients can obtain the best survival benefit from combination therapy. Forest plots of the subgroup analyses for OS and PFS indicated that the TACE+LEN + PD‐1 group provided a superior survival benefit in almost all subgroups analyzed compared with the TACE+LEN group, especially for Child–Pugh grade, PVTT classification, and tumor immune‐related cytokines, which were associated with prognosis. The presence of PVTT or Child–Pugh grade was identified as an independent prognostic factor for OS and PFS in our study, which is consistent with the results of previous studies.^38^, ^65^ In addition, patients with Child–Pugh grade B and VP3/VP4 PVTT had a significant response to the combination therapy of TACE+LEN + PD‐1 for OS compared with patients in the TACE+LEN group (HR <0.5). More notably, in subgroup analyses, the prolonged OS in patients treated with TACE+LEN + PD‐1 was not observed in patients with portal vein main invasion (VP4: mOS, 8.7 months), but it was observed in patients without portal vein main invasion (VP2: mOS, 28.6 months or VP3: mOS, 20.5 months), suggesting that the use of TACE+LEN + PD‐1 may be better in patients with HCC without portal vein main involvement than those with portal vein main involvement, thus improving the survival rate. Subgroup analyses also showed that patients with Child–Pugh grade A had better OS than patients with Child–Pugh grade B in the TACE+LEN + PD‐1 group (27.5 vs. 18.0 months, *p* = 0.0039). The formation of PVTT may cause portal vein obstruction, thus reducing liver blood flow perfusion, leading to deterioration of liver function, and TACE treatment further aggravates liver function damage. A study conducted by Fu et al.^66^ demonstrated that TACE combined with lenvatinib could prolong PFS, extend the interval between two TACE treatments, and lower the number of TACE sessions compared with TACE alone, thus preventing the deterioration of liver function usually caused by repeated TACE. In our study, the TACE treatment intervals of the TACE+LEN + PD‐1 and TACE+LEN groups were 81.35 ± 27.55 days and 63.72 ± 25.49 days (*p* = 0.013), respectively. Thus, Child–Pugh grade and PVTT classification might be noninvasive and predictive indicators to determine which type of patient can obtain the best survival benefit from combination therapy.

More importantly, ongoing transformation and clinical studies are expected to enable us to better understand tumor immune markers, gene mutations, and other factors that determine the response of HCC to immunotherapy. The PD‐1/PD‐L1 pathway is related to the occurrence of HCC, and its expression is associated with the high recurrence rate of tumors after surgery.^67^, ^68^ The immune checkpoint blockade of the PD‐1/PD‐L1 pathway and chimeric antigen receptor T‐cell and cytokine therapies has obtained promising outcomes. However, only a small proportion of patients with HCC benefit from these immunotherapy methods, and the low response of immunotherapy is partly due to the creation of an immunosuppressive tumor microenvironment and the reduction of T‐cell activity. The high proportion of circulating Tregs and MDSCs in HCC also implicates their potential role in HCC tumorigenesis.^69^ An immunosuppressive environment to promote tumor escape is created by enhancing the release of immunosuppressive cytokines, including IL‐5, IL‐8, and IL‐10, from activated immunosuppressive cells (Tregs and MDSCs) and inhibiting the release of immune‐activating cytokines, including IL‐1, TNF‐α/β, and IFN‐γ.^70^ It seems to emphasize the important role of cytokine signaling pathways in the occurrence and development of HCC. As described in the multivariate analysis, OS was significantly associated with IL‐6, IL‐17, IFN‐α, and VEGF levels, whereas PFS was significantly associated with IL‐6 and IFN‐α levels. A previous study^71^ also reported that IL‐6 level was a novel prognostic biomarker for patients with advanced HCC treated with Atezo/Bev, revealing significantly shorter PFS and OS periods in the IL‐6‐high group than in the IL‐6‐low group, which is consistent with our study in terms of OS by subgroup analyses (mOS, 25.5 vs. 19.6 months; *p* = 0.0373). This may be because the proinflammatory cytokine IL‐6 can recruit MDSCs, which inhibit T cells reactive to antitumor activity,^72^ suggesting that IL‐6 levels are positively correlated with HCC progression and negatively correlated with the antitumor immune response. The IL‐6/STAT3 signaling pathway is activated by IL‐17 and directly induces VEGF release to promote tumor angiogenesis.^73^, ^74^ Furthermore, according to the subgroup analyses, TACE+LEN + PD‐1 provided a better OS in low VEGF than in high VEGF levels (mOS, 26.3 vs. 17.6 months; *p* = 0.0042) and in low IL‐17 than in high IL‐17 levels (mOS, 24.7 vs. 20.0 months; *p* = 0.0196). Moreover, it may be of great significance to further investigate the possible coaction between the IL‐6/STAT3 signaling pathway and tumor immune efficacy in patients with HCC.

In addition, tumor‐associated macrophages (TAMs), the main type of inflammatory cells, inhibit antitumor immune responses by establishing an environment that promotes inflammation and tumor development, which are classified as the M2 phenotype.^75^ However, TAMs also differentiate into macrophages with antitumor properties, belonging to the M1 phenotype.^76^, ^77^ Reprogramming TAMs into M1 macrophages is considered a strategy to inhibit tumor growth and invasion, and IFN‐α treatment precisely exerts this antitumor effect in HCC.^78^ We found that patients with high IFN‐α levels had a more significant response related to longer OS compared with those with low IFN‐α levels (mOS, 26.0 vs. 18.5 months; *p* = 0.0219) in the subgroup analyses, indicating that IFN‐α might have a cooperative effect with TACE+LEN + PD‐1 for inhibiting HCC growth and invasion. Terawaki et al.^79^ demonstrated that IFN‐α was an innate source of feedback inhibition on Ag‐specific T cells through its direct activation of PD‐1 transcription in infection and antitumor immunity and that PD‐1 expression on Ag‐specific CD8^+^ T cells was augmented by IFN‐α in vivo. They demonstrated that IFN‐α administration in combination with PD‐1 blockade in tumor‐bearing mice effectively augmented antitumor immunity and proposed this as a novel and rational approach for cancer immunotherapy. To sum up, we identified and validated the fact that the inflammatory cytokines IL‐6, IL‐17, VEGF, and IFN‐α might be novel biomarkers for predicting the survival prognosis of patients with HCC and PVTT in triple combination treatment.

In our study, all AEs in both groups were controllable, and no treatment‐related deaths occurred in either group. The most frequent AEs were hand‐foot syndrome, hypertension, anorexia and nausea, fatigue, and hyperbilirubinemia. However, the most important grade 3–4 AEs included hand‐foot syndrome, hyperbilirubinemia, and thyroid dysfunction, which can be managed by drug reduction and symptomatic treatment. Additionally, the frequency and severity of AEs in the two groups were similar, suggesting that the administration of PD‐1 inhibitors does not significantly increase the risk of AEs in TACE combined with lenvatinib.

This study has some limitations. First, this was a retrospective study, with selection bias owing to the small sample size and single‐center design. Second, potential differences between patient options and baseline characteristics may affect treatment outcomes. Third, the results of subgroup analysis should be interpreted with caution. Consequently, future studies with large multicenter samples are required to validate the present results, and further studies should analyze the predictive markers of this combination therapy.

## 5 CONCLUSIONS

TACE+LEN + PD‐1 has markedly better outcomes than TACE+LEN in patients with HCC and PVTT, which is associated with the superiority of DCR, ORR, PFS, and OS and the manageable AEs in this study. OS is significantly correlated with Child–Pugh grade and PVTT classification, which may be noninvasive predictors for identifying patients who may benefit from combination therapy. Most importantly, inflammatory cytokines, including IL‐6, IL‐17, VEGF, and IFN‐α, might be novel biomarkers for predicting the survival prognosis of patients with advanced HCC and PVTT receiving TACE+LEN + PD‐1 treatment.

## AUTHOR CONTRIBUTIONS

**Xinhua Zou:** Conceptualization (equal); data curation (equal); formal analysis (equal); investigation (supporting); methodology (equal); project administration (supporting); resources (lead); software (lead); writing – original draft (lead). **Qingyu Xu:** Conceptualization (equal); project administration (lead); resources (equal); validation (equal); visualization (equal); writing – review and editing (lead). **Ran You:** Data curation (equal); formal analysis (equal); funding acquisition (equal); resources (equal); software (equal); supervision (equal); validation (equal). **Guowen Yin:** Conceptualization (equal); data curation (equal); funding acquisition (lead); investigation (lead); methodology (lead); project administration (lead); validation (equal); visualization (equal); writing – original draft (supporting); writing – review and editing (lead).

## FUNDING INFORMATION

This study was supported by the interventional radiology research project of Jiangsu Medical Association (1210012021081) and Jiangsu Province Excellent Postdoctoral Program (325825).

## CONFLICT OF INTEREST STATEMENT

The authors declare that they have no conflict of interest.

## ETHICS STATEMENT

This study was approved by the Ethics Committee of the Jiangsu Institute of Cancer Research (no. 202201032).

## INFORMED CONSENT STATEMENT

All patients provided written informed consent. This study was conducted in accordance with the principles of the Declaration of Helsinki.

## Data Availability

The datasets used in this study are available from the corresponding author upon reasonable request.
